# Prevalence of Antimicrobial Resistance and Infectious Diseases in a Hospitalised Migrant Population in Paris, France, a Retrospective Study

**DOI:** 10.3389/ijph.2022.1604792

**Published:** 2022-12-15

**Authors:** Sarah Stabler, Olivier Paccoud, Léa Duchesne, Marie-Aude Piot, Nadia Valin, Dominique Decré, Pierre-Marie Girard, Valérie Lalande, Karine Lacombe, Laure Surgers

**Affiliations:** ^1^ GHU APHP. Sorbonne Université, Service des Maladies Infectieuses et Tropicales, Hôpital Saint-Antoine, Paris, France; ^2^ Sorbonne Université, INSERM, Institut Pierre Louis d’Épidémiologie et de Santé Publique, Paris, France; ^3^ Département de Psychiatrie, Institut Mutualiste Montsouris, Paris, France; ^4^ Sorbonne Universités, Université Paris Descartes, UMR 1018/INSERM 1178, Centre de Recherche en Épidémiologie et Santé des Populations (CESP), Paris, France; ^5^ APHP, Hôpital Saint-Antoine, Département de Bactériologie, Paris, France; ^6^ Sorbonne Université, UPMC Univ Paris 06 CR7, INSERM U1135, CIMI, Team E13, Paris, France

**Keywords:** migrants, schistosomiasis, psychological disorders, AMR carriage, ESBL-E, sexual transmitted infections, drepanocytosis

## Abstract

**Objectives:** The aim of this study was to estimate the prevalence of anti-microbial resistance (AMR) carriage and its risk factors in hospitalized migrants. Additionally, the prevalence of infectious diseases was evaluated, as well as symptoms of psychological trauma.

**Methods:** We conducted a retrospective monocentric cross-sectional study including all migrant patients recently arrived and hospitalised over a one-year period.

**Results:** Among 101 patients, seventy-nine percent originated from Sub-Saharan Africa. The overall AMR carriage rate was 20.7% [95% CI: 12.4; 28.9%]. We isolated 5/92 methicillin-resistant *Staphylococcus aureus* strains (5.4%) and 15/92 extended-spectrum beta-lactamase-producing Enterobacteriaceae (16.4%). AMR carriage was associated with older age, region of origin and length of migration. Rates of HIV, HBV, and HCV infection were 39.6%, 32.7%, and 5%, reflecting sampling bias linked to reasons for hospitalization. Eleven percent had serological evidence of treponemasis and 7.8% had *Chlamydia trachomatis* infection. Symptoms of depression or post-traumatic stress disorder were observed for more than half the patients.

**Conclusion:** It appears essential to offer a systematic and comprehensive post-arrival screening of AMR carriage, infectious diseases and psychological trauma to subjects who experienced migration.

## Introduction

Emergence and dissemination of multi-drug resistant (MDR) and extensive drug-resistant (XDR) bacteria are an increasing public health problem, notably for extended-spectrum beta-lactamase producing Enterobacteriaceae (ESBL-E) [1]. International travels in particular play an important part in the worldwide spread of MDR bacteria [2]. According to the United Nations Migration Agency, we are facing a dramatic increase in the number of displaced people across various regions of the world. Since 2015, more than 2 million migrants have entered Europe during the recent refugee crisis [3].

Migrants are exposed to an increased risk of carriage of MDR bacteria and various other infectious diseases due to supposed higher prevalence rates in countries of origin [4], adverse migratory conditions (such as compromised hygiene and sanitation arrangements, overcrowding in containment areas) [5, 6], limited access to healthcare systems [7], and poor living conditions after resettlement [8]. Exposure to violence and trauma during migration, as well as difficult post-migratory living conditions, have been linked to serious psychological disorders in settled migrants [9, 10].

Few studies, summarised in a recent review [11], are available concerning the rate of anti-microbial resistance (AMR) carriage in newly-arrived migrants in Europe. Rates of MDR bacteria (ESBL-E and methicillin-resistant *Staphylococcus aureus* (MRSA)) carriage range between 20% [12, 13] and 60% [14] in Germany, and between 25% and 30% in Italy [15] and the Netherlands [16], respectively. No such data are available in France. Concerning infectious diseases, high rates of latent tuberculosis and hepatitis B infections have been reported in refugee and asylum seekers in various countries around Europe [17, 18]. In a recent French study performed by Médecins du Monde, rates of positive testing for HIV, HBV and HCV were respectively of 3.6%, 10.9%, and 8.6% in newly-arrived migrants. Syphilis testing was positive for 1.6% of patients and rates of *Chlamydiae trachomatis (CT)*, and *Neisseria gonorrhoeae* infections were of 6.4% and 1.6%, respectively [19]. The European Centre for Disease Prevention and Control (ECDC) recommends the screening of latent tuberculosis, HIV, HCV, HBV, schistosomiasis, and strongyloidiasis for newly-arrived migrants depending on their country of origin [20]. However, there is likely significant heterogeneity in screening practices across France and in Europe, which emphasises the need for additional data.

Lastly, literature is scarce concerning the mental health of migrants arriving in France. Studies suggest that exposure to traumatic experiences before and during migration and post-migration stress are associated with psychiatric disorders such as depression, anxiety, and post-traumatic stress disorder (PTSD) [9, 21]. In a systematic review performed in 2005, symptoms of PTSD and depression were reported in 9% and 5% of refugees, respectively [10], and more recent data are lacking.

The objectives of this study were 1) to estimate the rate of carriage of MDR and XDR bacteria, 2) to identify factors associated with AMR carriage, 3) to evaluate rates of HIV, HBV, HCV, syphilis, *Chlamydia trachomatis* (CT)*, Neisseria gonorrhoeae* (NG)*,* and schistosomiasis infections, 4) and to screen for history of physical or sexual abuse and symptoms evocative of depression or PTSD in hospitalised migrants in and out settings.

## Methods

### Patient Selection and Study Design

We performed a retrospective cross-sectional study that included patients who sought care in the Infectious Diseases department of a university teaching hospital (Saint-Antoine Hospital, Paris, France). Medical records of subjects hospitalised in in-patient and out-patient units from November 2017 to the end of October 2018 were screened for inclusion. All migrants, defined as 1) foreign-born and 2) having arrived in France within 12 months from Eastern Europa, Africa, the Middle-East, Asia or South-America were eligible. Non-inclusion criteria was age inferior to 18 years.

### Data Collection

Clinical and biological variables were retrospectively retrieved from our systematic post-arrival survey archived in the medical files (Supplementary Figure S1) and local computer healthcare systems and included [1]: demographic characteristics (age, gender, country of birth and living, marital status, profession, spoken language) [2], history of migration (date of departure and of arrival, countries travelled, means of travel, living conditions during travel (refugee camp, prison), history of violence (physical or sexual), living conditions in France, and administrative status [3]; medical history (including alcohol and tobacco consumption, prior vaccinations, prior antibiotic use, reason for hospitalisation) and symptoms evocative of psychiatric conditions including depression, anxiety, and PTSD using the standardize tool (Mini International Neuropsychiatric Interview (MINI) [4]; microbiological data, which are part of the hospital usual screening policy for migrant patients. Screening for MDR/XDR bacteria were performed with precultures in brain heart infusion broth with 10-µg/ml cefotaxime, 3.3-µg/ml ertapenem, or 3.3-µg/ml vancomycin (to select for ESBL-E, Carbapanemase-producing *Enterobacteriaceae* (CPE), or Vancomycin-resistant Enterococcus (VRE), respectively) after inoculation from nasal and anal swabs. Each culture were plated on medium for selective isolation (bioMerieux ESBL for ESBL-E). Bacterial colonies were identified using mass spectrometry (MALDI Biotyper; Bruker) and susceptibility testing used Mueller-Hinton agar (Bio-Rad). Serological testing were systematically performed for HIV (HIV-Antibody (Ab), HBV (HBs Antigen (Ag), HBc-Ab, HBs-Ab), HCV (HCV-Ab), syphilis (TPHA-VDRL) (Regent Kits Abbott) as well as PCR testing for CT and NG in urine or vaginal samplings (Roche Diagnostics). Hemoglobin electrophoresis and schistosomiasis serology (ELISA Novatec, Western Blot LDbio diagnostics) were also performed for all patients originating from Sub-Saharan Africa.

Data from de-identified patients were collected in a computerized database with a double data entry check.

### Statistical Analyses

As ESBL-E carriage was the most prevalent, statistical analyses focused on it. MRSA was not included because of different modes of transmission and risk factors. Demographic, migration, living conditions in France, clinical, and laboratory characteristics were compared between patients with or without ESBL-E carriage using the chi-squared or Fisher’s exact tests and the Student or Wilcoxon ranked tests for categorical and continuous variables, respectively. The association between ESBL carriage and the covariables was then examined using a multiple logistic regression model. A backward-stepwise process based on the Akaike information criterion was used to select the risk factors. Nine variables were included in this selection process: first, all covariables with a *p*-value <0.1 in univariate analysis, except the variables “diagnosis” and “time from arrival to sampling” (because of its similarity with the variable “time from arrival to hospitalization” and the absence of significant differences in the time from hospitalization and sampling between both groups) and, second, factors *a priori* deemed to be possibly significant risk factors (HBV and HIV infections). The final model included the 3 variables shown in the column «Multivariable analysis» of Table 3. ORs, 95% CIs and *p*-values were calculated. Significance was determined by a *p*-value <0.05. Statistical analyses were performed using R version 3.6.1.

## Results

**FIGURE 1:**
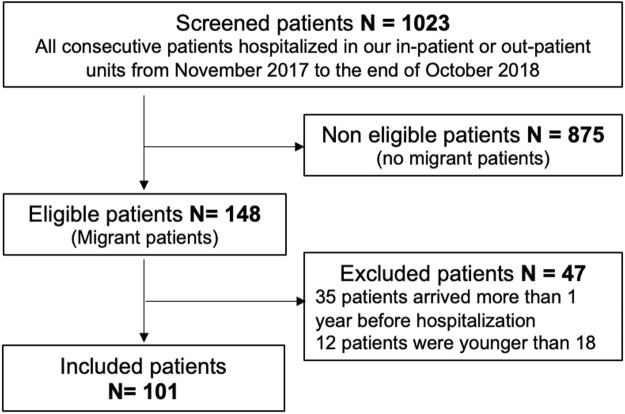
Flow chart (Migrinf study, France, 2018).

Over the one-year period, 1023 records were screened, and 101 patients were included in the present analysis after identification of 148 eligible patients and exclusion of 47 (Figure 1). Among these, 71 were male (70.3%), with a median age (IQR [Q1, Q3]) of 30 years (12 [24, 36]). Seventy-nine (78.2%) originated from Sub-Saharan Africa and 30 (30.9%) were asylum seekers. The principal routes of migration were by direct flight (n = 31, 32%), through Libya and Italy (*n* = 32, 33%), and through Morocco and Spain (*n* = 24, 24.7%). The median duration of migration (IQR [Q1, Q3]) was 21.7 weeks (70.4 [0.14, 70.5]), and 30.1% of patients had been in at least one refugee camp. In France, 65% lived in poor conditions (homelessness or living in temporary accommodations, including shelters, refugee camps or hostels) (Table 1).

**TABLE 1:** Patient characteristics (Migrinf study, France, 2018).

	n	Value
Demographics		
Gender, male, n (%)	101	71 (70.3)
Age (years), median (IQR)	101	30.0 (12.0)
Marital status, married, n (%)	98	24 (24.5)
Region of origin, n (%)	101	
Sub-Saharan Africa		79 (78.2)
South America		4 (4.0)
Asia		4 (4.0)
Eastern Europa		7 (6.9)
North Africa		3 (3.0)
Middle-east		4 (4.0)
Urban area, n (%)	89	26 (29.2)
Education level, no higher education, n (%)	93	68 (73.1)
Profession, n (%)	94	
Craftmanship		36 (38.3)
Higher occupation		7 (7.4)
Student		13 (13.8)
Intermediate occupation		14 (14.9)
No profession		24 (25.5)
Alcohol use, n (%)	98	15 (15.3)
Tobacco use, n (%)	99	19 (19.2)
Drug use, n (%)	96	3 (3.1)
Recent antibacterial therapy (previous 6 months), n (%)	84	26 (31.0)
Migration		
Route of Migration, n (%)	97	
Direct flight		31 (32.0)
Through Lybia and Italy		32 (33.0)
Through Morocco and Spain		24 (24.7)
Through the Balkans and Greece		9 (9.3)
Other		1 (1.0)
Duration of migration (weeks), median (iqr)	88	21.7 (70.4)
Refugee camp (at least once), n (%)	92	28 (30.4)
Travel by foot (at least one border), n (%)	94	10 (10.6)
Physical abuse, n (%)	96	41 (42.7)
Sexual abuse, n (%)	90	14 (15.6)
Experienced or witnessed a life-threatening event, n (%)	95	40 (42.1)
Living conditions in France		
Poor living conditions^a^, n (%)	100	65 (65.0)
No health insurance coverage, n (%)	100	55 (55.0)
Asylum seeker, n (%)	97	30 (30.9)
Hospital data		
Reason for admission, n (%)	96	
Referral by a third party		68 (70.8)
Own consultation		5 (5.2)
Emergency consultation		23 (24.0)
In-patient hospitalisation, n (%)	101	43 (42.6)
Diagnosis, n (%)	100	
Bacterial infection		12 (12.0)
Viral infection		62 (62.0)
Tuberculosis		13 (13.0)
Other		13 (13.0)

^a^ Poor living conditions: homelessness or living in temporary accommodations, including shelters, refugee camps or hostels.

### AMR Carriage and Risk Factors for ESBL-E Carriage

The overall rate of carriage of MDR bacteria was 20.7% [95% CI: 12.4; 28.9%]. We isolated 5/92 (5.4%) MRSA strains and 15 (16.3%) ESBL-E, including 11 *Escherichia coli*, 3 *Klebsiella pneumoniae* and 1 *Enterobacter cloacae* (Tables 2, 3)*.* Of the 15 ESBL, 14 were CTX-M-15 enzymes and one was CTX-M-14. Neither carbapenem-resistant Enterobacteriaceae, imipenem-resistant *Acinetobacter baumanii* nor vancomycin-resistant *Enterococci* were isolated.

**TABLE 2:** Prevalence of anti-microbial resistance carriage, infectious diseases, and others (Migrinf study, France, 2018).

	Total (N)	Value	Estimated prevalence (%, 95% CI)
Overall AMR carriage, n (%)	92	19 (20.7)	20.7 (12.4–28.9)
MRSA carriage, n (%)	92	5 (5.4)	5.4 (0.8–10.0)
ESBL carriage, n (%)	92	15 (16.3)	16.3 (8.7–23.8)
IRAB, n (%)	92	0 (0)	—
CPE, n (%)	92	0 (0)	—
HCV infection, n (%)	100	5 (5.0)	5.0 (0.73–9.3)
HIV infection, n (%)	101	40 (39.6)	39.6 (30.1–49.1)
HBV infection, n (%)	101	33 (32.7)	32.7 (23.5–41.8)
Syphilis, n (%)	90	10 (11.0)	11.0 (4.6–17.4)
Schistosomiasis, n (%)	73	11 (15.1)	15.1 (6.9–23.3)
*Chlamydiae trachomatis*, n (%)	77	6 (7.8)	7.8 (1.8–13.8)
*Neisseria gonorrhoeae*, n (%)	77	0 (0)	—
*Mycoplasma spp*, n (%)	44	2 (4.5)	—
Haemoglobin electrophoresis, n (%)	58		—
Heterozygous sickle cell disease		6 (10.3)	10.3 (2.5–18.2)
Normal		45 (77.6)	77.6 (66.8–88.3)
Other abnormalities		7 (12.1)	12.1 (3.7–20.4)
Symptoms of depression^a^, n (%)	94	50 (53.2)	53.2 (43.1–63.3)
Symptoms of anxiety disorder^b^, n (%)	91	55 (60.4)	60.4 (50.4–70.5)

^a^ Sadness, low spirits, lack of willpower.

^b^ Excessive irritability, acute anxiety attack, chronic excessive anxiety and revivification episodes.

MRSA, methicillin resistant *Staphylococcus aureus*; ESBL, Extended Spectrum Beta-lactamase producing Enterobacteriaceae; IRAB, imipenem resistant *Acinetobacter Baumanii*; CPE, carbapenemase - producing Enterobacteriaceae.

**TABLE 3:** Factors associated with extended-spectrum beta lactamase producing Enterobacteriaceae (ESBL-E) carriage (Migrinf study, France, 2018).

	Total	ESBL -	ESBL +	Univariable analysis	Multivariable analysis	*p*-value
	(n)	(n = 77)	(n = 15)	*p*-value	OR (95% CI)^a^	
Demographics						
Gender, male, n (%)	92	56 (72.7)	9 (60)	0.322		
Age, median (IQR)	92	29.8 (9.9)	39.9 (12.7)	**< 0.001**	**22.2 (3.37–523.0)** ^b^	**0.008**
Region of origin, n (%)	92			**0.050**		
Sub-Saharan Africa		62 (80.5)	10 (66.7)			
South America		4 (5.2)	0 (0)			
Asia		3 (3.9)	0 (0)			
Eastern Europa		3 (3.9)	3 (20)			
North Africa		1 (1.3)	2 (13.3)			
Middle East		4 (5.2)	0 (0)			
Urban area, n (%)	80	48 (71.6)	11 (84.6)	0.496		
Profession, n (%)	85			0.093		
Craftmanship		26 (36.1)	5 (38.5)			
Higher occupation		6 (8.3)	1 (7.7)			
Student		12 (16.7)	0 (0)			
Intermediate occupation		8 (11.1)	5 (38.5)			
No profession		20 (27.8)	2 (15.4)			
Education level, no higher education, n (%)	84	52 (73.2)	9 (69.2)	0.765		
Alcohol use, n (%)	89	12 (16)	1 (7.1)	0.683		
Tobacco use, n (%)	90	15 (19.7)	1 (7.1)	0.449		
Drug use, n (%)	87	2 (2.7)	1 (7.1)	0.413		
Recent antibacterial therapy (previous 6 months) n (%)	76	18 (29)	6 (42.9)	0.334		
Migration						
Route of migration, n (%)	89			0.252		
Direct by flight		21 (28)	8 (57.1)			
Through Lybia and Italy		28 (37.3)	2 (14.3)			
Through Morocco and Spain		18 (24)	3 (21.4)			
Through the Balkans and Greece		7 (9.3)	1 (7.1)			
Other		1 (1.3)	0 (0)			
Direct route of migration	89	21 (28)	8 (57.1)	**0.033**		
Duration of migration (weeks), median (IQR)	79	25.3 (73.1)	0.4 (7.8)	**0.074**		
Refugee camp (at least once), n (%)	84	23 (32.9)	2 (14.3)	0.213		
Travel by foot (at least one border), n (%)	86	9 (12.5)	1 (7.1)	1.000		
Physical violence, n (%)	87	34 (46.6)	4 (28.6)	0.214		
Sexual violence, n (%)	82	10 (14.7)	2 (14.3)	1.000		
Living conditions in France						
Poor conditions of living, n (%)	91	48 (63.2)	11 (73.3)	0.647		
No social rights, n (%)	91	38 (50%)	12 (80%)	**0.064**		
Hospital data						
Way of hospital admission, n (%)	87			0.257		
Referral by a third party		52 (72.2)	9 (60)			
Own consultation		5 (6.9)	0 (0)			
Emergency consultation		15 (20.8)	6 (40)			
Inpatient hospitalisation, n (%)	92	32 (41.6)	10 (66.7)	0.133		
Time from arrival to sampling (days), median (IQR)	86	139.5 ± 176.8	38.0 ± 78.2	**0.005**		
Time from hospitalization to sampling (days), median (IQR)	86	1.0 ± 7.2	1.0 ± 3.5	0.612		
Time from arrival to hospitalisation (days), median (IQR)	86	121.5 ± 153.8	35.5 ± 81.2	**0.007**	0.991 (0.979–0.999)	0.108
Diagnosis, n (%)	91			**0.011**		
Bacterial infection		6 (7.9)	4 (26.7)			
Viral infection		50 (65.8)	7 (46.7)			
Tuberculosis		13 (17.1)	0 (0)			
Other		7 (9.2)	4 (26.7)			
HIV infection, n (%)	92	29 (37.7)	9 (60.0)	0.108	8.75 (0.519–419.5)	0.182
HCV infection, n (%)	91	2 (2.6)	3 (20.0)	**0.029**		
HBV infection, n (%)	92	27 (35.1)	2 (13.3)	0.132		

Bold values correspond to values <0.1 in the univariate.

^a^ Only the variables selected by the backward-stepwise process are shown.

^b^ For the multivariate analysis, 86 observations were included. The “age” variable was transformed into a binary variable, using the median age of the global population (i.e., 30 years old) as a threshold, as well as the « region of origin » variable (Sub-Saharan Africa versus other regions). 86 observations.

In univariate analysis, patients with ESBL-E carriage were significantly older (36 years-old vs. 28 years-old, *p* < 0.001) and originated from Eastern Europe (20% vs*.* 3.9%, *p* = 0.05) and North Africa (13.3% vs*.* 1.3%, *p* = 0,05). Patients with ESBL-E carriage more often arrived from direct flight (*p* = 0.033) and after a shorter duration of migration (0.4 weeks vs. 25.3 weeks, *p* = 0.074). The delay from arrival to hospitalization and sampling was shorter for patients with ESBL-E carriage (35 vs. 121 days, *p* = 0.007 and 38 vs. 139 days, *p* = 0.005 respectively). In multivariate analysis, only older age remained independently statistically associated with ESBL-E carriage (*p* = 0.008).

Older age, direct flight and shorter delay from arrival to sampling were also significantly associated with ESBL-E carriage when considering only patients originated from sub-Saharan Africa (data not shown).

### Prevalence of Other Infections and Others

Rates of HIV, HBV, and HCV infection were 39.6%, 32.7%, and 5% respectively. These high rates were reflecting sampling bias linked to reasons for hospital admission as patients were addressed for treatment and/or monitoring of these chronic viral infections (Table 2). Eleven out of 73 patients (15%) had serological evidence of schistosomiasis: nine were male and all originated from Sub-Saharan Africa. Six out of 77 (7.8%) had CT infection corresponding to 3 men and 3 women with a mean age of 28 years old. Hemoglobin electrophoresis performed in patients originating from sub-Saharan Africa showed abnormalities in almost a quarter (22.4%) of cases, including 10.3% of sickle cell disease trait. History of physical abuse during migration was reported in 41 patients (42.7%). Sexual abuse was reported by 12 women (42.9%). Fifty patients presented with symptoms suggesting depression (53.2%), and 55 presented with symptoms consistent with an anxiety disorder (60.4%).

## Discussion

In this cross-sectional study of migrant individuals coming to France mainly from Sub-Saharan Africa, MDR bacteria carriage was estimated at 20.7% [95% CI: 12.4; 28.9%]. To our knowledge, this is the first such study to be carried out in France and reporting the prevalence of AMR in migrants. AMR carriage in the general French population has been estimated around 6% in 2011 [22]. Recently-arrived migrants therefore seem to have higher AMR carriage rates than the French population. This higher rate has already been described in other European countries, and mostly ranged between 20 and 30% [12, 15, 16].

E-ESBL were the most frequent MDR bacteria isolated (16.3%), as previously reported [11]. To our knowledge, our study is the first to identify ESBL with all but one enzyme being CTX-M-15, confirming the spread and globalization of CTX-M enzymes and especially CTX-M-15 [23].

One of the most interesting results of this study is the significantly higher risk of ESBL-E carriage in cases of direct migratory trajectory by flight (*p* = 0.033) and a trend towards a higher risk in cases of shorter duration of migration (*p* = 0.074). This suggests that the country of origin may be an important marker for AMR carriage in newly-arrived migrants. The importance of the country of origin was previously described in a study in Switzerland, where a higher risk of ESBL-E carriage for patients was reported in patients originating from the Middle East [24]. However, a recent meta-analysis of previous European studies highlighted higher AMR prevalence in high-migrant community settings (refugee centers or outpatient’s clinics), suggesting that AMR carriage was mostly acquired during transit or in migrant settings [11]. Indeed, in the literature, adverse migratory conditions such as compromised hygiene and overcrowding in containment areas have been linked to increasing infectious diseases transmission [6]. Thus, both countries of origin and migratory conditions may have a role in AMR carriage acquisition and transmission.

We also found that older age was significantly associated with ESBL carriage, which to our knowledge has not been previously reported. As the patterns of migration in Europe are likely to evolve over time, with an expected generalization of migration from populations of more diverse age groups, this aspect certainly warrants further consideration.

High rates of venereal diseases such CT infections were found in our study (7.8% [95% CI: 1.8–13.8%]). It should however be noted that this could be confounded by similarly high rates of HIV infection in our population, due to bias caused by reasons for hospital referral. It can also be mentioned that CT infections were screened in vaginal or urinal samples and not in pharyngeal or rectal ones that may have led to underestimate CT prevalence. However, similar rates (6.4%) were previously reported in a study of newly-arrived migrants tested on a voluntary basis [19]. These rates remain high compared to the 3.8% globally observed in 2016 [25]. Screening for venereal diseases is not recommended for newly-arrived migrants. Our data suggest that it should be considered for systematic screening.

Serological evidence for schistosomiasis was found in 15% of patients originating from Sub-Saharan Africa. This high prevalence is similar to the 17% already reported in Italy [26], and strongly suggests the need for systematic screening in these populations. A recent European review confirmed the safety, high effectiveness, and cost-effectiveness of systematic screening of schistosomiasis and praziquantel-based treatment for newly-arrived migrants from endemic countries [27].

Hemoglobin electrophoresis was systematically performed in patients originating from sub-Saharan Africa and showed abnormalities in almost a quarter (22.4%) of cases, including 10.3% of sickle cell disease trait. In France, high-risk population could benefit from early diagnosis in order to prevent complications. Testing for sickle cell disease trait in migrants originated from Sub-Saharan could also lead to information about prenatal diagnosis. No data seemed available on this topic in the literature.

The high rates of psychological symptoms are a significant concern. Indeed, symptoms of depression and anxiety (from excessive irritability to PTSD) were reported in 53.2% [95% CI: 43.1–63.3%] and 60.4% [95% CI: 50.4–70.5%] of patients, respectively. This is higher than was previously reported in the literature: from 5 to 30% for depression and from 10 to 30% for PTSD [10, 28]. Psychological symptoms may be explained by pre-displacement factors, including political persecution, gender, and sexual orientation, and by post-displacement factors, such as social isolation and difficulties in finding employment and housing [29]. Once diagnosed, providing access and continuity to appropriate mental health care for these patients remains a significant challenge, due in part to infrequent recourse to healthcare professionals, lack of social security coverage, and to cultural considerations. Psychiatric symptoms in this setting are however barriers to social integration [30], and are likely to have long-term repercussions on the overall health of migrants. This highlights the urgent need for increased medical awareness of these issues and improvements in the screening and care for mental health in migrants.

The main limitations of our study are the small sample size and the retrospective nature that implies some missing data. Due to the low sample size of our study and the number of missing data for some co-variables, our study may be underpowered and therefore, have underestimated the prevalence of studied conditions and the association between ESBL carriage and some exposure variables. All the screening tests are routinely performed and correspond to the usual practice of our department, which minimizes the risk for recruitment bias and limits the amount of missing data. It is important to notice that the patients included in our study were specifically referred to our department for medical care, and therefore are not representative of the general migrant population. However, reasons for hospitalization were not directly related to the studied conditions which minimizes the risk for recruitment bias. Furthermore, as recommended in France (High Council of Public Health 2019), AMR carriage research was performed as soon as the patients are hospitalized in order to reduce any over risk directly related to hospital settings. Screening patients in first referral medical centers would improve the sensitivity of these analyses.

The European Center for Disease Prevention and Control recently published guidelines on screening for infectious diseases in newly-arrived migrants [20]. This study adds necessary data concerning the health of recently-arrived migrants. As high rates of MDR carriage, of CT infections, and of psychological disorders were observed in our population, the results of this study should argue for the addition of these conditions to a comprehensive screening policy of all newly-arrived migrants.

Finally, more comprehensive data regarding the global prevalence of AMR carriage are required, particularly in low-income countries where increased use of antibiotics is likely to increase the prevalence the prevalence of MDR bacteria. This would allow for a better understanding of the dynamics of dissemination of AMR carriage on a global scale and mitigate the risk of the spreading of an AMR pandemic.

### Conclusion

With regards to the high prevalence of AMR, infectious diseases and psychological disorders found in this population of migrants, it appears essential to offer a systematic and comprehensive post-arrival screening of infectious diseases and signs of psychological trauma to all subjects who experienced migration.

## Supplementary Material

Supplementary Figure S1 Example of systematic survey (France, 2018).
