# Supplementary figures and images for: Prevalence of Antimicrobial Resistance and Infectious Diseases in a Hospitalised Migrant Population in Paris, France, a Retrospective Study
Source: Int J Public Health. 2022 Dec 15;67:1604792. doi: 10.3389/ijph.2022.1604792 (PMC9797533; doi:10.3389/ijph.2022.1604792)

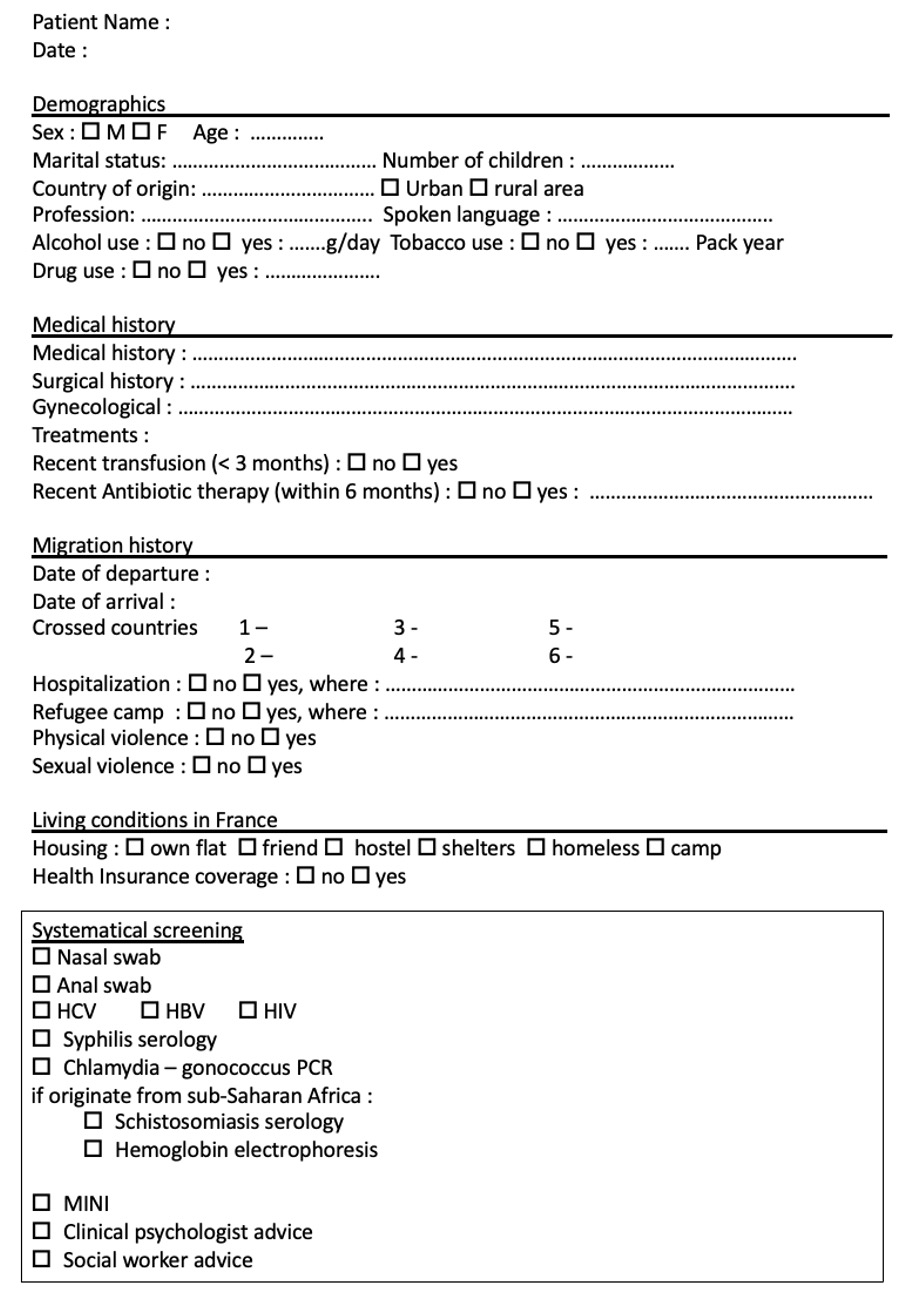

Supplement: Supplementary file 1 [file Image1.JPEG]
